# Short-term intermittent cigarette smoke exposure enhances alveolar type 2 cell stemness via fatty acid oxidation

**DOI:** 10.1186/s12931-022-01948-4

**Published:** 2022-03-02

**Authors:** Hidehiro Irie, Mari Ozaki, Shotaro Chubachi, Ahmed E. Hegab, Akihiro Tsutsumi, Naofumi Kameyama, Kaori Sakurai, Shingo Nakayama, Shizuko Kagawa, Sachika Wada, Makoto Ishii, Tomoko Betsuyaku, Koichi Fukunaga

**Affiliations:** Division of Pulmonary Medicine, Department of Medicine, Keio University School of Medicine, 35 Shinanomachi, Shinjuku-ku, Tokyo, 160-8582 Japan

**Keywords:** Intermittent cigarette smoke, Lung stem cell, Alveolar type 2 cell, Colony-forming efficiency, Fatty acid oxidation, Carnitine palmitoyltransferase 1a

## Abstract

**Background:**

Cigarette smoke (CS) is associated with chronic obstructive pulmonary disease (COPD) and cancer. However, the underlying pathological mechanisms are not well understood. We recently reported that mice exposed to long-term intermittent CS for 3 months developed more severe emphysema and higher incidence of adenocarcinoma than mice exposed to long-term continuous CS for 3 months and long-term continuous CS exposure activated alveolar stem cell proliferation. However, the influence of variations in the CS exposure pattern in alveolar stem cell in unknown. Here, we exposed mice to 3 weeks of continuous or intermittent CS to identify whether different CS exposure patterns would result in differential effects on stem cells and the mechanisms underlying these potential differences.

**Methods:**

Female mice expressing GFP in alveolar type 2 (AT2) cells, which are stem cells of the alveolar compartment, were exposed to mainstream CS via nasal inhalation. AT2 cells were collected based on their GFP expression by flow cytometry and co-cultured with fibroblasts in stem cell 3D organoid/colony-forming assays. We compared gene expression profiles of continuous and intermittent CS-exposed AT2 cells using microarray analysis and performed a functional assessment of a differentially expressed gene to confirm its involvement in the process using activator and inhibitor studies.

**Results:**

AT2 cells sorted from intermittent CS-exposed mice formed significantly more colonies compared to those from continuous CS-exposed mice, and both CS-exposed groups formed significantly more colonies when compared to air-exposed cells. Comparative microarray analysis revealed the upregulation of genes related to fatty acid oxidation (FAO) pathways in AT2 cells from intermittent CS-exposed mice. Treatment of intermittent CS-exposed mice with etomoxir, an inhibitor of the FAO regulator Cpt1a, for 5 weeks resulted in a significant suppression of the efficiency of AT2 cell colony formation. In vitro treatment of naïve AT2 cells with a FAO activator and inhibitor further confirmed the relationship between FAO and AT2 stem cell function.

**Conclusions:**

Alveolar stem cell function was more strongly activated by intermittent CS exposure than by continuous CS exposure. We provide evidence that AT2 stem cells respond to intermittent CS exposure by activating stem cell proliferation via the activation of FAO.

**Supplementary Information:**

The online version contains supplementary material available at 10.1186/s12931-022-01948-4.

## Introduction

Lung cancer and chronic obstructive pulmonary disease (COPD) cause significant morbidity and mortality worldwide [1]. Emphysema is the irreversible destruction of distal airspaces and is an independent risk factor for lung cancer [2, 3]. Cigarette smoke (CS) is the major inducing factor for both lung cancer and COPD [4, 5]. Nevertheless, the mechanisms underpinning CS-induced emphysema and cancer have not been fully elucidated. We previously optimized an animal model of CS-induced lung cancer and emphysema. Using this model, we demonstrated that long-term continuous CS exposure for 3 months caused mild emphysema-like changes in the lung. Further, exposure to the same amount of CS in an intermittent manner resulted in a more severe form of emphysema and substantial induction of lung adenocarcinoma [6]. The intermittent CS exposure pattern induced marked changes in lung inflammatory cells underscored by an increase in macrophages, particularly M2-polarized macrophages within and around the vicinity of the adenocarcinomas [6].

Alveolar type 2 (AT2) cells play a major role in lung homeostasis and serve as a stem cell pool for self-regeneration and Alveolar type 1 (AT1) cell regeneration following alveolar injury [7, 8]. Long-term CS exposure is known to cause chronic inflammation, oxidative stress, and apoptosis of alveolar epithelial cells, which results in the destruction of the lung matrix [9]. We previously demonstrated that long-term CS exposure increased the number of AT2 cells, activated their stem cell function, and induced resistance to apoptosis, which was in contrast to our hypothesis that long-term CS exposure would suppress AT2 stem cell activity [10]. Mechanistically, we provided evidence that long-term CS exposure induced perturbations in circadian genes, thus inducing AT2 cell proliferation and resistance to apoptosis [10]. Based on our results in these two long-term CS exposure studies, we hypothesized that short-term (3 weeks) CS exposure would also induce AT2 stem cell activation. The aim of this study was to investigate whether different CS exposure patterns (continuous versus intermittent) would result in differential effects on AT2 stem cells and the mechanisms underlying these potential differences. To this end, we exposed two mouse groups to 3 weeks of either continuous or intermittent CS and examined AT2 stem cell activity using our in vitro 3D organoid/colony-forming assay [11]. We compared gene expression profiles of continuous and intermittent CS-exposed AT2 cells. Finally, we performed a functional assessment of a differentially expressed gene to confirm its involvement in the process using activator and inhibitor studies, and examined the effects on alveolar stem cell activation in a 3D organoid/colony-forming assay.

## Materials and methods

### Mice

Mice expressing green fluorescent protein (GFP) in cells expressing surfactant protein C (Sftpc) (CBA/Ca × C57BL6J) were obtained from Brigid Hogan, Duke University [12]. In this mouse line, GFP is strongly expressed in all AT2 cells and weakly expressed in a small population of cells at the bronchioalveolar duct junction (BADJ) and proximally located terminal bronchioles [11]. C57BL6J mice were used when GFP fluorescence was not required. All animal experiments were reviewed and approved by the Institutional Animal Care and Use Committee at Keio University.

### CS exposure

**Fig. 1:**
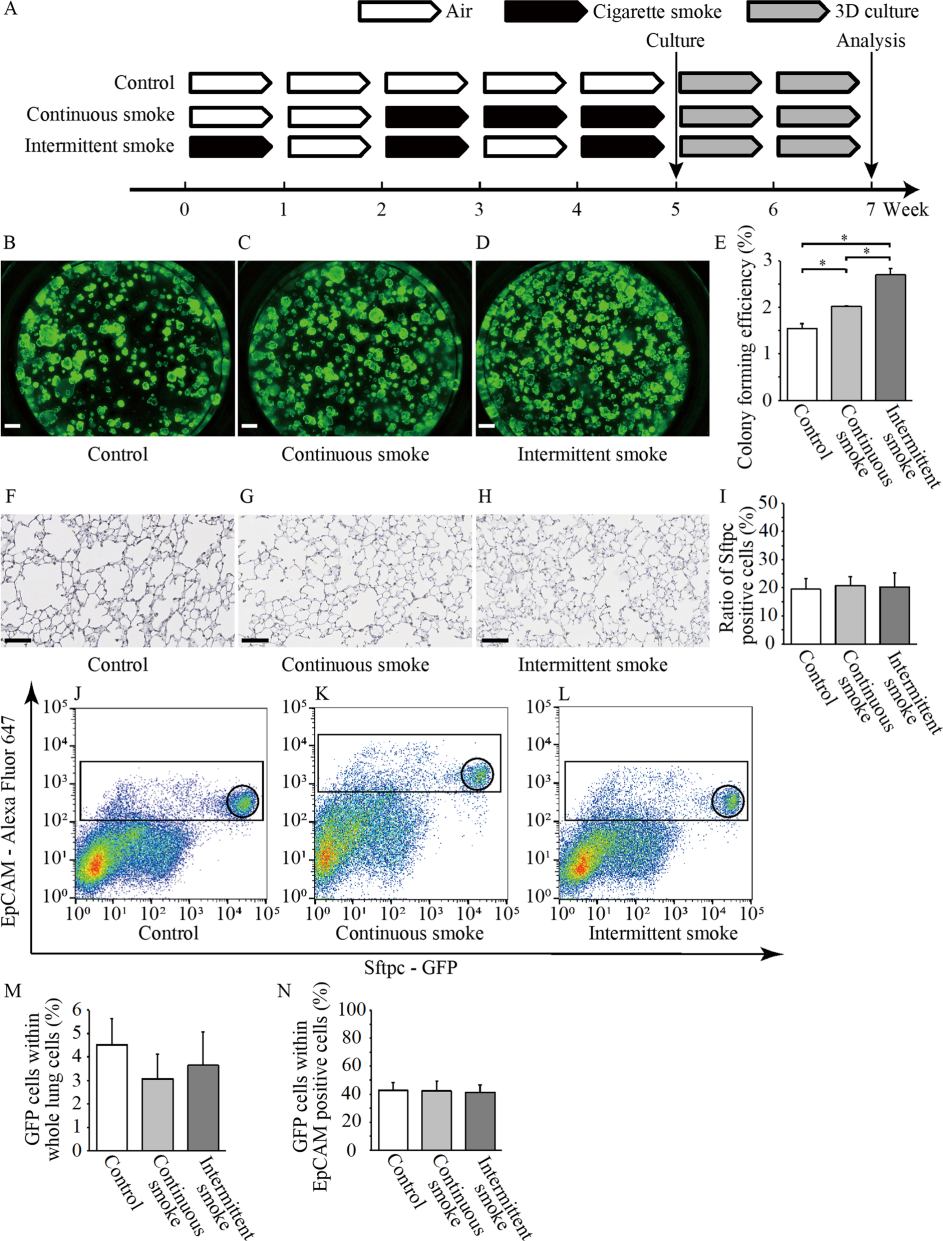
Effect of short-term intermittent cigarette smoke (CS) exposure on alveolar stem cells. **A** Study design and experimental protocol. Adult Sftpc/GFP and C57BL6 mice were exposed to CS using this regimen. **B**–**E** Effect of different CS exposure protocols on AT2 stem cell function as determined with a colony-forming assay. Sorted EpCAM^high^/GFP^high^ cells were cultured in an in vitro three-dimensional (3D) stem cell colony-forming assay. Representative day 14 images are presented for air-exposed (**B**), continuous CS-exposed (**C**), and intermittent CS-exposed alveolar colonies (**D**), demonstrating that colony-forming efficiency (CFE) of continuous CS-exposed AT2 cells was higher than that of air-exposed AT2 cells, and CFE of intermittent CS-exposed AT2 cells was higher than that of air-exposed and continuous CS-exposed AT2 cells. Quantification of CFE was conducted using triplicate wells (**E**). Data are presented as means ± SD. The reproducibility was confirmed from three independent experiments. *P < 0.05. Scale bars: 500 μm. **F**–**I** Whole lung sections from air-exposed and CS-exposed mice were immunohistochemically stained for Sftpc to identify AT2 cells. Sftpc-positive cells in the alveolar areas of air-exposed mice (**F**), continuous CS-exposed mice (**G**), and intermittent CS-exposed (**H**) mice were quantified for the percentage of Sftpc-positive cells from each group (**I**). Data are presented as means ± SD. N = 7–8 per group. *P < 0.05. Scale bars: 100 μm. **J**–**L** The proportion of AT2 cells within whole lung cells (WLCs) and epithelial cells. Dissociated WLCs were stained with EpCAM antibody and examined by flow cytometry. Representative flow cytometry dot plot for the WLCs of air-exposed (**J**), continuous CS- exposed (**K**), and intermittent CS-exposed (**L**) mice. **M** and **N** Percentage of GFP^high^ (i.e., AT2) cells within WLCs (**M**) and EpCAM^high^ (i.e., epithelial) cells (**N**) within the lungs of air-exposed, continuous CS-exposed, and intermittent CS-exposed mice. Data are presented as means ± SD. Data were compiled from three repeated experiments

Female Sftpc/GFP and C57BL6 mice (9–11 weeks old) were exposed to mainstream CS generated from commercially available filtered cigarettes (Marlboro, 12 mg tar/1.0 mg nicotine) via nasal inhalation as previously reported [13]. To test the effects of repeated cycles of CS exposure on AT2 cell behavior, mice were divided into three groups. The study design and experimental protocol are presented in Fig. 1A. The mice were exposed to CS for 60 min/day, 5 days/week, and were sacrificed 24 h after the last CS exposure. Age-matched control mice were exposed to air over the same time period.

### Immunohistochemistry and immunofluorescence

Paraffin-embedded lungs and colony blocks were freshly sectioned (6 μm) and stained with hematoxylin and eosin (H&E) and cell-type specific antibodies. The primary antibodies used were goat and rabbit anti-Sftpc (Santa Cruz Technology, Dallas, TX; Millipore, Billerica, MA), mouse anti-PCNA (Abcam, Cambridge, UK), rabbit anti-*aquaporin-5 (A*qp5) (Abcam), mouse anti-E-cadherin (*BD, Bedford, MA*), rabbit anti-vimentin (Cell Signaling Technologies, Danvers, MA), and rabbit anti Cpt1a (Proteintech, Rosemont, IL; Invitrogen, Carlsbad, CA). The appropriate horse radish peroxidase-enzyme or Alexa-Fluor-conjugated secondary antibodies (Invitrogen, Carlsbad, CA) were used. Nuclei were counterstained with hematoxylin or DAPI (Vector Labs, Burlingame, CA) and examined. Cell numbers were quantified from histological images by visual counting from at least seven random images per group.

### Bronchoalveolar lavage and glutathione analysis

The lungs of 6–7 mice per group were lavaged with 0.6 mL of saline three times using a tracheal cannula [6]. Total cell counts of the bronchoalveolar lavage (BAL) fluid (BALF) were determined, and cell differentials in BALF were examined as previously described [6, 14]. BALF supernatants were used for measurement of glutathione (GSH) and glutathione disulfide (GSSG) using the Cayman Chemical Glutathione Assay Kit (Cayman Chemical, Ann Arbor, MI) according to the manufacturer’s protocol. GSH and GSSG concentrations were calculated using a standard curve and normalized to total protein levels in each sample [15].

### Lung digestion and collection of lung epithelial cells by FACS

Lung digestion and the collection of different cell types were conducted as previously described [11]. In brief, lung lobes were collected from two or three mice per group, were pooled together and finely minced with scissors. These samples were then incubated in a liberase (Roche, Madison, WI)/dispase (BD) enzyme mixture for 10 min at 37 °C. The solution was then passed through a 21G needle four times and filtered using a 100 μM mesh. DNase (Worthington, Lakewood, NJ) was added to the mesh to help release entangled cells. Red blood cells were lysed using Ammonium-Chloride-Potassium (ACK) lysis buffer. Whole lung cells (WLCs) were sorted on a MoFlo XDP (Beckman Coulter, Brea, CA) for EpCAM^high^/GFP^high^; i.e., AT2 cells. AT2 cells were used for in vitro culture or for preparation of cytospin slides (2.5 × 10^4^ cells) for staining with rabbit anti-Aqp5 (Abcam) (a marker for AT1 cells) and Cpt1a (the rate-limiting enzyme for long chain fatty acid oxidation) [16]. WLCs separated into single cells using the method above and AT2 cells were used for cytospin slides (2.5 × 10^4^ cells) for staining with mouse anti-8-hydroxy-2′-deoxyguanosine (8-OHdG) (Abcam), rabbit anti-γ-H2AX (Abcam), rabbit anti-Aqp5 (Abcam) (a marker of AT1 cells), Cpt1a, and DAPI (CA). For fibroblast collection, hematopoietic and endothelial cells were depleted by staining the cells with CD31 and CD45 MACS^®^ MicroBeads followed by sorting using MACS^®^ (Miltenyi Biotec, Bergisch Gladbach, Germany). CD45^−^CD31^−^ cells were incubated in RPMI (Invitrogen) supplemented with 10% fetal bovine serum (FBS) at 37 °C, 5% CO_2_, and 95% air, in T75 flasks overnight. Attached cells were considered to be fibroblasts.

### in vitro 3D organoid colony-forming assay

Lung fibroblasts (1.0–2.0 × 10^4^ cells) were co-cultured with the same number of sorted EpCAM^high^/GFP^high^ cells in a 2:1 growth factor-reduced Matrigel^®^ (BD Biosciences, San Jose, CA). A volume of 150 μL was placed in each transwell (CORNING, Corning, NY). MTEC/Plus medium (600 μL) was added to the lower chamber and replaced every 3–4 days. The number of colonies per insert was counted on days 12–16. Both fluorescence and phase-contrast images were obtained using a Keyence BZ-X810 microscope. The number of colonies was quantified by visual counting as previously described [11]. Collection of the Matrigel 3D colonies and processing for histological examination were performed as described previously [11].

### Administration of etomoxir to mice

Treatment was performed via intraperitoneal administration of etomoxir (inhibitor of the carnitine palmitoyltransferase (Cpt1) enzyme; Sigma-Aldrich, St. Louis, MO; 25 mg/kg) or vehicle (normal saline) every other day for 5 weeks to intermittent CS-exposed, continuous CS-exposed and air-exposed mice [17]. Lung digestion, collection, and in vitro 3D organoid colony forming assay was conducted as described above. The number of colonies per insert was counted on days 12 to 16.

### Treatment of 3D cultures with etomoxir and L-carnitine

Lung fibroblasts (1.0–2.0 × 10^4^ cells) were co-cultured with the same number of sorted EpCAM^high^/GFP^high^ cells in a 2:1 growth factor-reduced Matrigel^®^ (BD Biosciences). A volume of 150 µL was placed in each transwell. MTEC/Plus medium and etomoxir (0, 50, 100, and 150 μM, diluted in phosphor-buffered saline; PBS) or L-carnitine (FUJIFILM Wako Pure Chemical Corporation, Tokyo, Japan) (0, 1, and 10 mM, diluted in MTEC/Plus medium) were added to the lower chamber and replaced every other day. The volume of the lower chamber was adjusted to 600 µL with MTEC/Plus medium and the etomoxir solution or L-carnitine solution. The number of colonies per insert was counted on days 12–16.

### Microarray analysis

For each group, AT2 cells were pooled together from three mice, and used for microarray analysis. Total RNA was extracted from sorted these EpCAM^high^/GFP^high^ cells using TRIzol reagent (Invitrogen) according to the manufacturer’s protocol. RNA integrity was examined using an Agilent 2100 BioAnalyzer (Agilent Technologies, Santa Clara, CA). Biotinylated ss-cDNA was prepared according to the standard Affymetrix protocol from 100 ng total RNA using the GeneChip WT PLUS Reagent Kit User Manual (Affymetrix/Thermo Fisher Scientific, Waltham, MA). Fragmented and labeled ss-cDNA was hybridized on a GeneChip Clariom S Array (Affymetrix/Thermo Fisher Scientific). Scanned output files were analyzed using the Transcriptome Analysis Console (Affymetrix/Thermo Fisher Scientific). Microarray data sets were deposited in the National Center for Biotechnology Information Gene Expression Omnibus under accession number GSE162919.

### Real-time PCR

Total RNA was extracted from sorted EpCAM^high^/GFP^high^ cells or WLCs using TRIzol reagent or RNeasy Mini Kit (Qiagen, Venlo, Netherlands) according to the manufacturer’s protocol. cDNA was synthesized from total RNA using the High Capacity RNA-to-cDNA kit (ThermoFisher Scientific). The reverse transcription-polymerase chain reaction was prepared using the SYBR FAST ABI Prism qPCR Kit (Kapa Biosystems, Wilmington, MA) according to the manufacturer’s protocol. Gene expression levels were analyzed using the QuantStudio 5 Real-Time PCR System (ThermoFisher Scientific). Mouse beta actin was used as the endogenous control for normalization. The primers used were listed in Additional file 5: Table S1.

### Statistical analysis

Data are expressed as means ± standard deviation (SD). In the 3D organoid/colony-forming assay, AT2 cells were collected from two or three mice per group. Quantification of colony formation was conducted using triplicate wells, and the reproducibility was confirmed from at least two repeated experiments. Data were compared using the two-tailed Student’s *t*-test or ANOVA, followed by the Tukey–Kramer test. Categorical data were analyzed using the χ^2^ test. Results were considered statistically significant at P < 0.05. All data were analyzed using JMP, version 15.0 (SAS Institute, Cary, NC).

## Results

### Short-term intermittent exposure to CS induced alveolar stem cell function

We previously reported that exposure to 3 months of intermittent CS caused more severe emphysematous changes in the lungs in mice when compared to continuous exposure [6] and that 3 months of CS exposure activated alveolar stem cell proliferation [10]. In this study, we aimed to investigate the effects of short-term (3-week) continuous versus intermittent CS exposure. Mice were exposed to air, continuous CS, or intermittent CS for 3 weeks and then sacrificed. GFP-positive AT2 stem cells were sorted from the lungs, and stem cell activation status was examined in an in vitro colony-forming assay (Fig. 1A). This assay enables direct comparison of stem cell behavior (number of activated stem cells within a cell population and their proliferation and differentiation) by seeding a large number of lung epithelial cells in a 3D matrix in co-culture with niche cells, which mimics the in vivo environment while permitting for quantitative observation of stem cell behavior [11]. Compared to air-exposed cells, continuous CS-exposed AT2 cells formed significantly higher numbers of colonies (Fig. 1B–E). Furthermore, intermittent CS-exposed AT2 cells formed significantly higher numbers of colonies when compared to the continuous CS-exposed cells (Fig. 1B–E).

To identify whether the differences reflect the effect of chronic smoking exposure or acute smoking exposure, we performed an experiment to compare the number of colonies between the two groups at 1 week, 2 weeks, 3 weeks, 4 weeks and 5 weeks, and quantified the colony number ratio of the intermittent smoke group/the continuous smoke group (Additional file 1: Fig. S1A). Intermittent CS-exposed AT2 cells formed significantly higher numbers of colonies when compared to the continuous CS-exposed cells at 1 week, 2 weeks, 3 weeks, and 5 weeks. However, there was no difference in the number of colonies between the two groups at 4 weeks. These results indicate that even one week of acute smoking exposure increases the number of colonies, and that although CS exposure periods were similar between the two group at 4 weeks and 5 weeks, intermittent CS exposure increases the number of colonies more than continuous smoking exposure (Additional file 1: Fig. S1B, C).

**Fig. 2:**
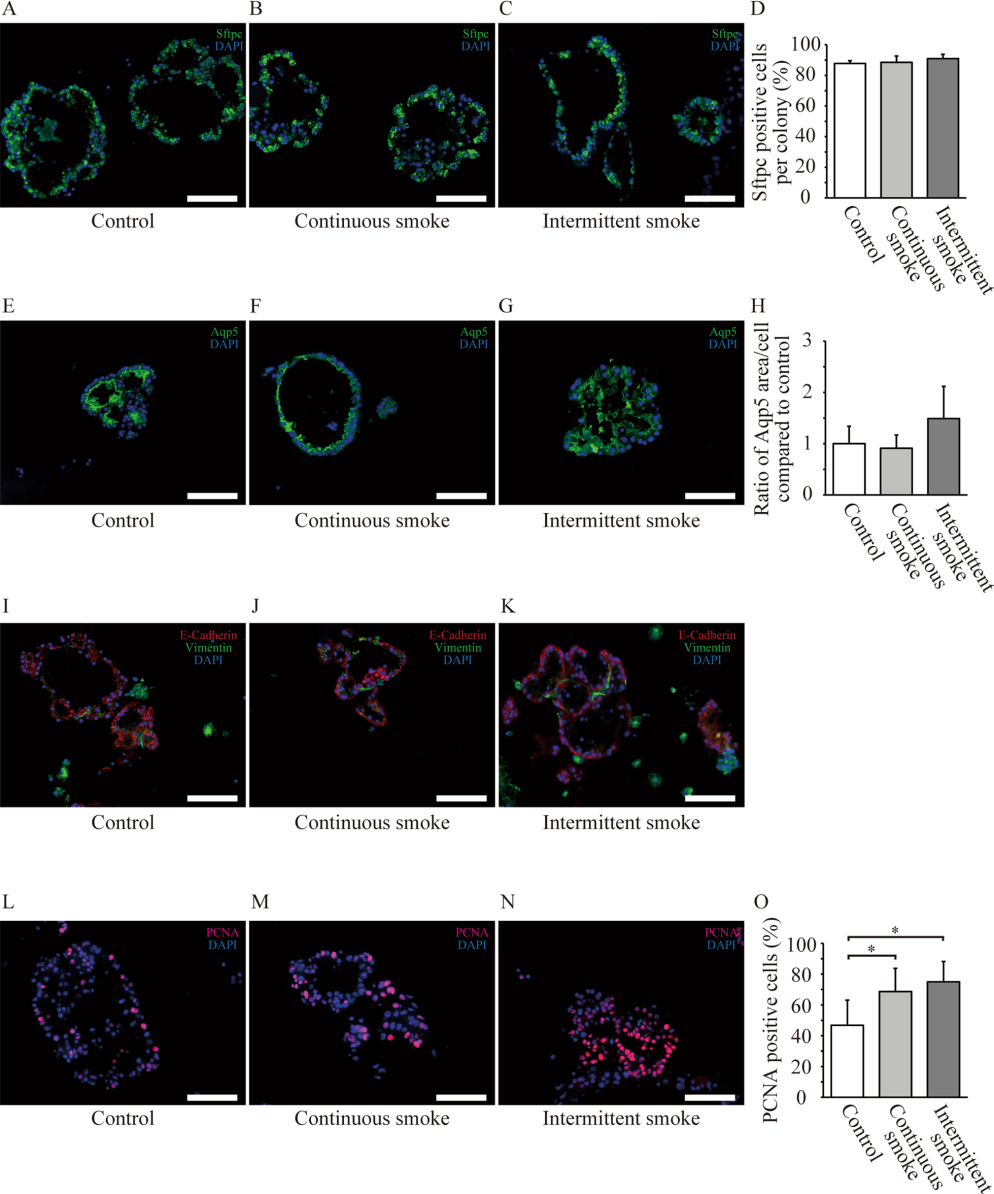
Immunofluorescent characterization of growing colonies. **A**–**D** Representative images depicting staining for surfactant protein c (Sftpc) in air-exposed (**A**), continuous cigarette smoke (CS)-exposed (**B**), and intermittent CS-exposed (**C**) mice. No significant difference was noted in the ratio of the number of Sftpc-positive cells in colonies among the three groups. Data are presented as means ± SD. N = 4–5 per group. Scale bars: 100 μm. **E**–**H** Representative images depicting staining for aquaporin-5 (Aqp5) in air-exposed (**E**), continuous CS-exposed (**F**), and intermittent CS-exposed mice (**G**). No significant difference was observed in the ratio of Aqp5-stained area per cell among the three groups (**H**). The ratio in each group was calculated based on the average value of the air-exposed group. Data are presented as means ± SD. N = 5–6 per group. Scale bars: 100 μm. **I**–**K** Colonies immunofluorescence-stained for E-cadherin and vimentin. Scale bars: 100 μm. **L**–**N** Representative images depicting staining for PCNA in air-exposed (**L**), continuous CS-exposed (**M**), and intermittent CS-exposed mice (**N**). Scale bars: 100 μm. **O** Comparison of PCNA positive cell ratio in colonies growing in 3D culture in air-exposed, continuous CS-exposed, and intermittent CS-exposed mice. Data are presented as means ± SD. N = 8 (air), 13 (continuous CS exposure), and 5 (intermittent CS exposure)

We previously demonstrated that mouse lungs exposed to 3 months of continuous CS contained significantly higher numbers of AT2 cells when compared to air-exposed lungs [10]. To identify whether these effects were present from as early as 3 weeks of CS exposure, we stained and quantified the AT2 marker, Sftpc, in histological lung sections from the three groups. No significant difference was observed in the number of Sftpc-positive cells among the three groups (Fig. 1F–I). No significant differences were observed in the percentage of GFP-positive (AT2) cells within the WLCs and lung epithelial cells collected from intermittent CS-exposed, continuous CS-exposed, and air-exposed control mice (4.52 ± 1.10%, 3.06 ± 1.05%, and 3.64 ± 1.41%, respectively; P = 0.38; 42.92 ± 5.57%, 42.41 ± 7.25%, and 41.43 ± 5.18%, respectively; P = 0.95; Fig. 1J–N). These results indicated that alveolar stem cell function was more strongly activated by intermittent CS exposure than by continuous CS exposure, but both exposure patterns were insufficient to cause significant increases in the number of AT2 cells within the lungs. To further characterize the effects of intermittent CS exposure on AT2 stem cells, we collected growing colonies from all groups at 14 days and stained histological sections for Sftpc and Aqp5, which are markers of AT2 and AT1 cells, respectively. No significant difference was observed in the ratio of AT1 and AT2 cells within the various colonies from all groups, indicating that both continuous and intermittent CS exposure did not perturb the ability of AT2 progenitors to differentiate into AT1 cells when compared to naïve control cells (Fig. 2A–H). Colonies formed by continuous and intermittent CS-exposed AT2 cells exhibited tight wrapping with vimentin-positive fibroblasts, resembling observations of naïve air-exposed control AT2 cells (Fig. 2I–K). Significantly higher numbers of PCNA-positive cells were observed in colonies from continuous and intermittent CS-exposed AT2 cells than in colonies from air-exposed cells. No significant difference was observed between colonies from continuous and intermittent CS-exposed AT2 cells (Fig. 2L–O).

### Enhanced self-renewal caused by intermittent CS exposure did not involve oxidative stress and inflammation

As oxidative stress has been reported to be involved in the self-renewal of stem cells and the onset of lung cancer [18, 19], we conjected that it could be involved in the enhanced self-renewal of alveolar stem cells in the intermittent CS exposure group. We first examined whether the expression of antioxidant genes was influenced by CS exposure pattern. qPCR analysis using whole lung RNA revealed that continuous CS exposure caused a significant increase in the expression of several of the genes examined, whereas intermittent CS exposure resulted in a significant reduction in the expression of all the antioxidant genes examined when compared to controls (Additional file 2: Fig. S2A–D). We next examined the expression of 8-OHdG, a marker of oxidative stress, and γH2AX, a marker of DNA damage, in whole-lung cells on cytospin slides. We observed that the ratio of 8-OHdG-positive cells was higher in the continuous CS exposure group than in the control and intermittent CS exposure groups (Additional file 2: Fig. S2E–H), but no significant differences were observed in the ratio of γH2AX-positive cells among the three groups (Additional file 2: Fig. S2I–L).

We subsequently analyzed glutathione metabolism in the BALF. Glutathione functions as an electron donor or acceptor by cycling between reduced (GSH) and oxidized (GSSG) forms and plays a crucial role in xenobiotic detoxification, protein folding, antioxidant defense, and other processes [20, 21]. No significant differences were observed in the concentrations of GSH, GSSG, GSH + GSSG, and the ratio of GSSG/GSH among groups (Additional file 2: Fig. S2M-P). Given the well-established involvement of inflammation in lung cancer and CS-induced lung emphysema, we examined the BALF inflammatory profile of air-exposed, continuous CS-exposed, and intermittent CS-exposed mice. Total cell number and the numbers of macrophages and neutrophils in BALF were significantly higher in continuous CS-exposed mice than in intermittent CS-exposed and air-exposed mice (Additional file 3: Fig. S3A–C). These results indicated that oxidative stress and inflammation were mostly attenuated by intermittent CS exposure compared to continuous CS exposure.

### Transcriptional profile of AT2 cells was altered in response to intermittent CS exposure

**Fig. 3:**
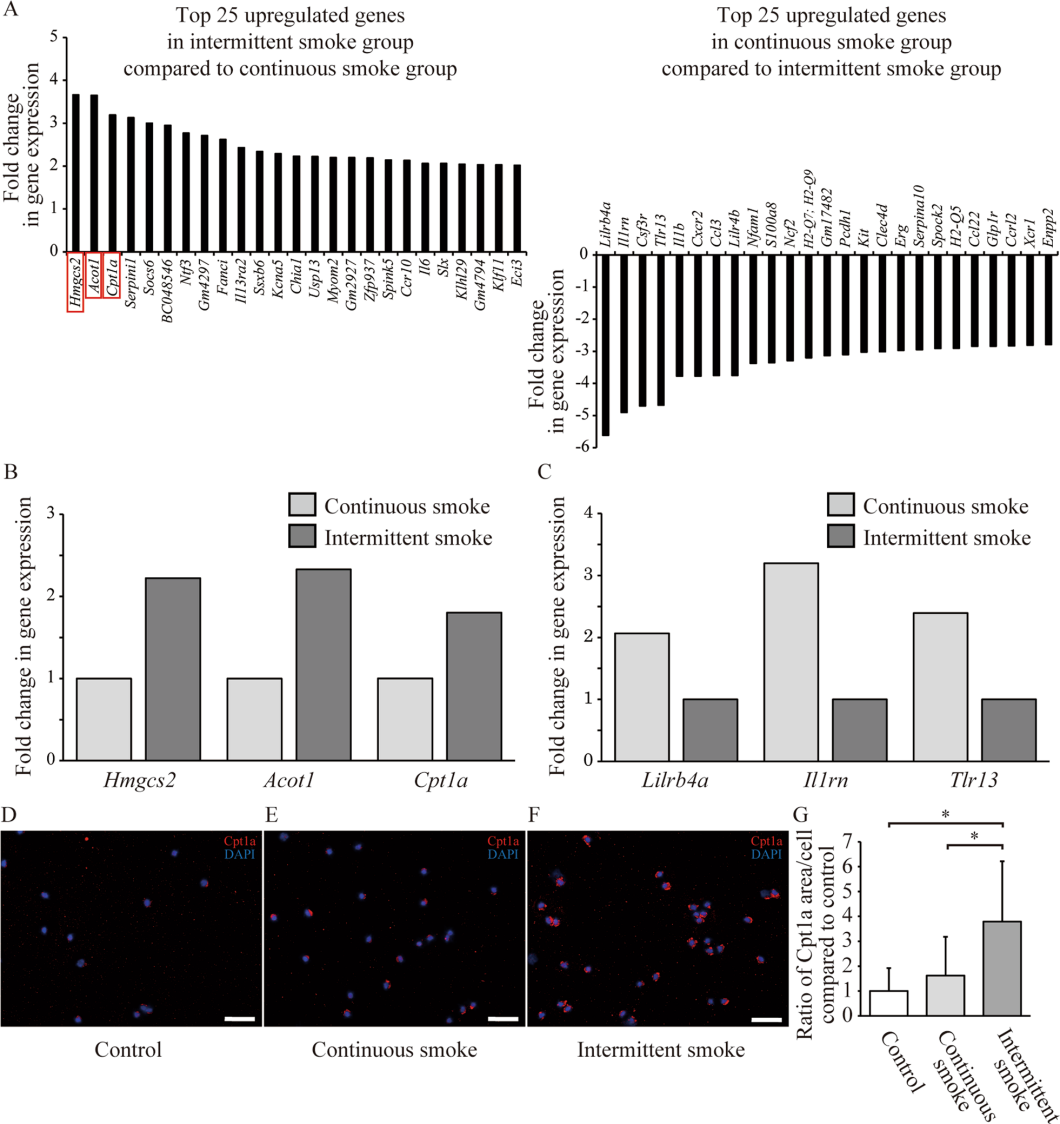
Differentially regulated genes in AT2 cells exposed to continuous and intermittent CS. For each group, AT2 cells were pooled together from three mice, and used for microarray analysis and real-time PCR. **A** Top 25 upregulated and downregulated genes identified in the microarray analysis in continuous CS-exposed and intermittent CS-exposed groups. The top three genes in the intermittent CS-exposed group (red boxes) are related to fatty acid metabolism. **B** and **C** Confirmation of several of the upregulated genes by real-time PCR. Data are presented as relative expression compared to levels in AT2 cells isolated from continuous CS-exposed or intermittent CS-exposed lungs. **D**–**G** Representative images of cells immunofluorescence-stained for Cpt1a in cytospin slides from air-exposed (**D**), continuous CS-exposed (**E**), and intermittent CS-exposed groups (**F**). The ratio of Cpt1a-stained area per cell was significantly higher in the intermittent CS-exposed group than in the air-exposed and continuous CS-exposed group. The ratio in each group was calculated based on the average value of the air-exposed group. Data are presented as means ± SD. N = 35 (air), 52 (continuous CS exposure) and 89 (intermittent CS exposure). *P < 0.05. Scale bars: 50 μm

RNA samples from AT2 cells sorted from at least three mice per group were analyzed using an Affymetrix microarray. We observed 26 genes that were upregulated and 141 that were downregulated by at least two-fold in the intermittent CS-exposed group when compared to continuous CS-exposed AT2 cells (Fig. 3A). The validity of the array results was confirmed via several approaches. First, reverse transcription-polymerase chain reaction amplification of the RNA samples for several up- and downregulated genes confirmed their enrichment/reduction in intermittent CS-exposed AT2 cells (Fig. 3B, C). Second, genes that are known to be specifically expressed in AT2 cells were enriched in the array results (e.g., *Sftpc, Sftpa1, Sftpb, Sftpd, Lamp3, Lcn2, Lyz1,* and *Lyz2*). Functional category analysis of the 26 upregulated genes in AT2 cells exposed to intermittent CS revealed an enhancement of fatty acid metabolism pathways, as the most strongly upregulated genes were *Hmgcs2, Acot1,* and *Cpt1a* (red boxes in Fig. 3A). Genes involved in the negative regulation of peptidase activity were also upregulated, whereas genes related to cell chemotaxis, positive regulation of cytosolic calcium ion concentration, neutrophil degranulation, positive regulation of neuron apoptotic processes, hematopoietic cell lineage, learning or memory, and regulation of vasculature development were downregulated. The microarray results revealed that many inflammation-related factors (*IL-1b, Cx3cl1, Cxcl2, Ccl3, Ccl22, Ccl6, Xcr1, Ccr1, Cxcr2,* and *Cxcr4*) were upregulated in continuous CS-exposed AT2 cells when compared to intermittent CS-exposed AT2 cells, consistent with the results from the BALF cell counts. No differences were detected in the expression of antioxidant genes such as *Gpx1, Gpx2, Gpx3, Gpx4, Gclc, Hmox1, Hmox2, Nqo1, Nqo2,* and *Gsr* between air-exposed, continuous CS-exposed, and intermittent CS-exposed cells.

### Fatty acid oxidation mediated the intermittent CS exposure-induced enhancement of AT2 stem cell self-renewal

**Fig. 4:**
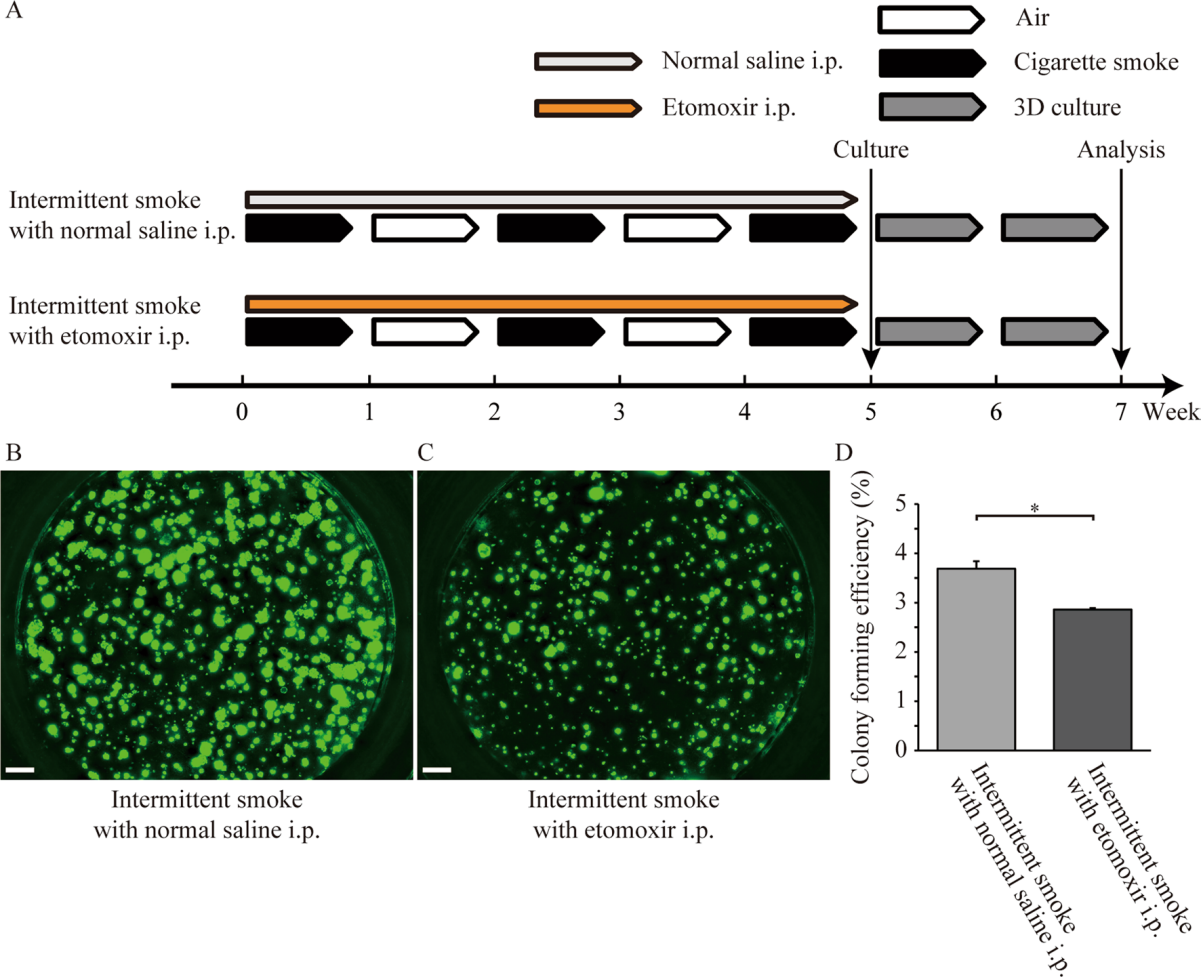
Effect of blocking FAO on intermittent CS-induced activation of AT2 stemness. **A** Study design and experimental protocol. The carnitine palmitoyltransferase (Cpt1), inhibitor etomoxir (25 mg/kg) or vehicle (normal saline) was intraperitoneally injected into intermittent CS-exposed Sftpc/GFP mice every other day for 5 weeks. **B**–**D** Representative day 14 images are presented for intermittent CS-exposed mice treated with normal saline i.p. (**B**) and etomoxir i.p. (**C**). Colony-forming efficiency (CFE) was inhibited in the etomoxir group (**D**). Quantification of CFE was conducted using triplicate wells. Data are presented as means ± SD. The reproducibility was confirmed using two repeated experiments. *P < 0.05. Scale bars: 500 μm

Previous studies have demonstrated the critical involvement of fatty acid oxidation (FAO) in regulating the function of intestinal [22] and neural stem cells [23]. Among the FAO critical regulators, activity of Cpt1a was demonstrated to be a major contributor to intestinal stem cell proliferation [22]. As *Cpt1a* was one of the most strongly upregulated genes in AT2 stem cells in response to intermittent CS exposure, we investigated whether FAO mediated the enhancement of AT2 cell stemness induced by intermittent CS exposure. We first compared the expression of Cpt1a by immunostaining in sorted AT2 cells and observed that Cpt1a expression was significantly higher in intermittent CS-exposed AT2 cells than in continuous CS-exposed and air-exposed AT2 cells (Fig. 3D–G). Next, we administered etomoxir, an irreversible inhibitor of Cpt1a [22, 24], intraperitoneally to block FAO in vivo in mice exposed to continuous or intermittent CS for 5 weeks and examined whether this treatment reversed the enhancement of in vitro AT2 stem cell colony formation compared to that of saline-treated, continuous or intermittent CS-exposed AT2 cells (Fig. 4A and Additional file 4: Fig. S4A). Etomoxir treatment of continuous CS-exposed mice showed no effect on the colony-forming efficiency of AT2 stem cells compared to that in cells collected from saline-treated mice (Additional file 4: Fig. S4B–D). However, etomoxir treatment of intermittent CS-exposed mice significantly suppressed the colony-forming efficiency of AT2 stem cells compared to that in cells collected from saline-treated mice (Fig. 4B–D). This suggested that intermittent CS-induced enhancement of AT2 stem cell function involved the activation of FAO and Cpt1a, and pharmacological inhibition of FAO abrogated this intermittent CS-induced enhancement.

**Fig. 5:**
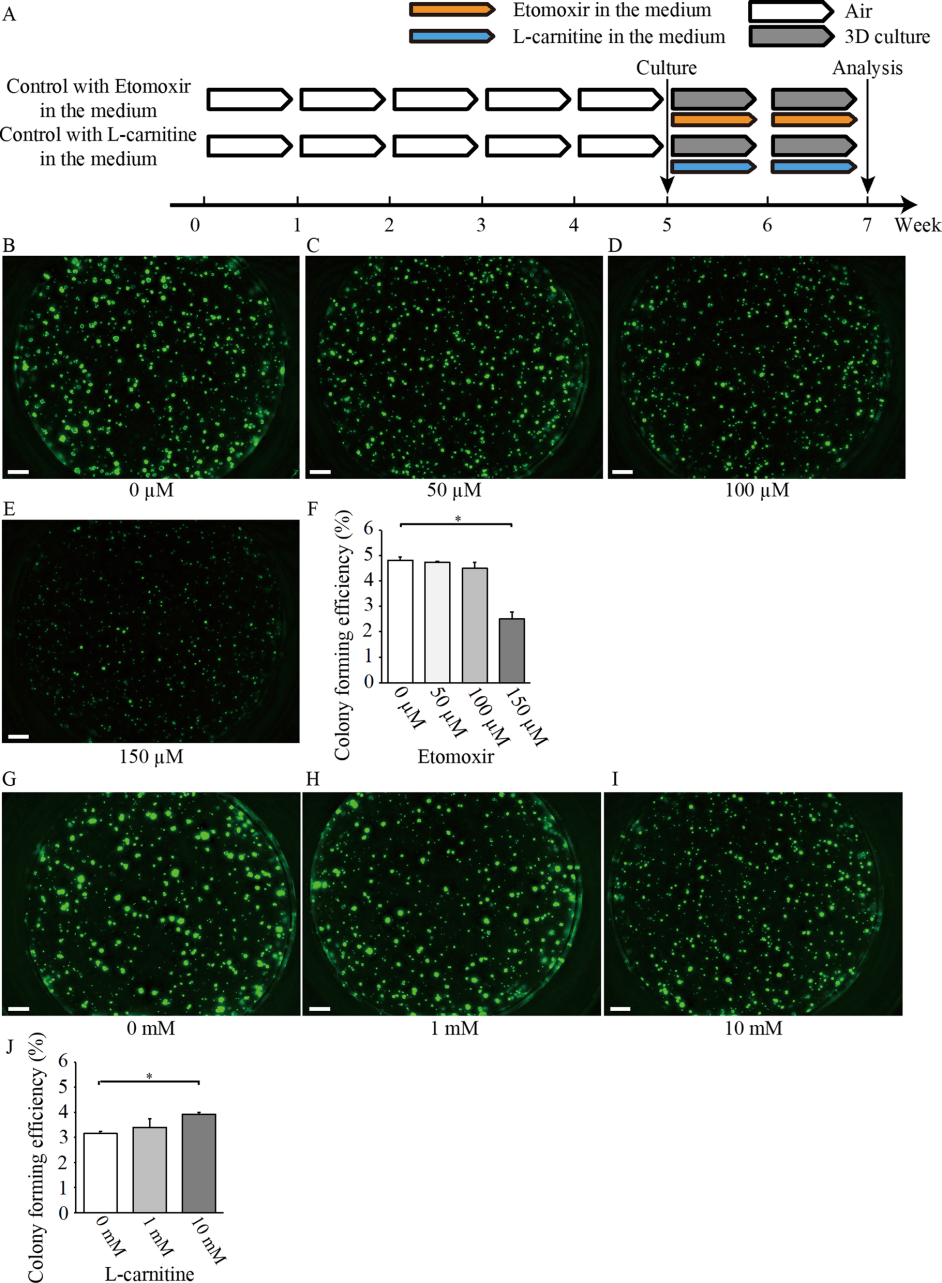
Effect of blocking or inducing FAO in vitro on naïve AT2 stem cells. **A** Study design and experimental protocol. The effect of different etomoxir or L-carnitine concentrations on colony formation in the 3D colony-forming assay was evaluated in air-exposed mice. Etomoxir or L-carnitine was added to the medium. (B-E) Representative day 14 images of alveolar colonies treated with different concentrations of etomoxir are presented. **F** Comparison of the effect of etomoxir. Quantification of colony-forming efficiency (CFE) was conducted using triplicate wells. Data are presented as means ± SD. The reproducibility was confirmed using two repeated experiments. **G**–**I** Representative day 14 images are presented for alveolar colonies treated with different concentrations of L-carnitine. **J** Comparison of the effect of L-carnitine. Quantification of CFE was conducted using triplicate wells. Data are presented as means ± SD. The reproducibility was confirmed using two repeated experiments. *P < 0.05. Scale bars: 500 μm

To further confirm the effects of FAO on alveolar stem cell activation independent to CS exposure, we treated AT2 cells sorted from air-exposed control mice with etomoxir or L-carnitine (a metabolite critical for transporting long-chain fatty acids into the mitochondria for subsequent β-oxidation; i.e., it enhances FAO [25] and upregulates *Cpt1a* mRNA expression [26] in vitro), and examined the effects on AT2 cell colony-forming efficiency (Fig. 5A). Treatment with etomoxir significantly suppressed AT2 cell colony-forming efficiency at the 150 µM concentration (Fig. 5B–F). In contrast, treatment with L-carnitine significantly upregulated AT2 cell colony-forming efficiency at the 10 mM concentration (Fig. 5G–J). Collectively, these data suggest that FAO is directly implicated in the regulation of AT2 stem cell function.

## Discussion

We recently reported that long-term (3-month) continuous CS exposure enhanced AT2 stemness in a murine model [10]. Here, we demonstrate that even relatively short-term continuous CS exposure enhanced the stemness of AT2 cells, and this enhancement was further augmented with an intermittent pattern of CS exposure. Previous studies have reported that intermittent irritation causes more harmful effects when compared to continuous irritation in several murine models. For example, intermittent hypoxia mimicking obstructive sleep apnea promoted lung tumorigenesis [27] and accelerated tumor progression when compared to chronic hypoxia [28]. Further, the mechanism of lung damage caused by intermittent hypoxia involved inflammation and oxidative stress [29]. We thus hypothesized that intermittent CS exposure would cause cytotoxicity and lung inflammation, resulting in greater DNA damage and compensatory cell proliferation, when compared to continuous CS-exposure. Contrary to our hypothesis, we observed that inflammation was more pronounced in continuous CS-exposed mice than in intermittent CS-exposed mice. Additionally, no differences in oxidative stress were detected between the two CS-exposed groups.

AT2 cells constitute the main epithelial stem cell pool of the alveolar compartment [30]. To maintain homeostasis, AT2 cells slowly self-renew and differentiate over a 1-year period but undergo rapid cycles of proliferation and differentiation following injury [30]. Several studies have reported the involvement of Wnt and bone marrow protein 4 (BMP4) signaling pathways in AT2 stem cell regulation. Wnt signaling is associated with smoke-induced diseases [31, 32]. However, neither Wnt nor BMP were differentially regulated between continuous and intermittent CS-exposed groups in our experiments. CS exposure is considered a lower grade injury compared to other injury models such as bleomycin and pneumonectomy. The Responses of AT2 cells vary by injury, which has been demonstrated in upper airway basal stem cells in which the pathways involved differ according to injury type [8].

In our previous report using a chronic CS exposure mouse model, chronic CS exposure enhanced the stemness of AT2 cells via circadian rhythm-related genes but not via other genes previously reported as regulators of AT2 cell stemness [10]. In this study, we observed that intermittent CS exposure enhanced the stemness of AT2 cells via FAO activation but not via circadian rhythm-related genes as was observed for continuous CS exposure [10]. It is notable that different CS exposure protocols enhanced AT2 stemness via distinct mechanisms. Recent reports have demonstrated that AT2 cells are heterogeneous, and certain subpopulations of AT2 cells proliferate and differentiate into AT1 cells following severe injury [31, 33]. Our study did not identify which subpopulation of AT2 cells exhibited enhanced stemness in response to intermittent CS exposure, which is an issue worthy of further investigation.

Mitochondrial FAO is a major catabolic process that degrades long-chain fatty acids and has widespread effects on the regulation of cell fate in endothelial cells, immune cells, and cancer cells [34]. CS exposure has been reported to alter FAO in AT2 cells. Agarwal et al. demonstrated that short-term CS exposure altered glycolysis and increased FAO in AT2 cells [35], but the effect of different CS exposure protocols on AT2 stemness was unknown. Recent reports have highlighted the importance of FAO in stem cell maintenance. Fasting activates the FAO program, and acute genetic disruption of Cpt1a abrogated the enhancing effects of fasting on intestinal stem cells [22]. In neural stem cells, Cpt1a-dependent FAO was required for stem cell maintenance and proper neurogenesis [23]. In this study, we demonstrated that intermittent CS exposure activated the FAO program, and activation of the FAO program at least partly mediated the enhancement of AT2 cell stemness induced by intermittent CS exposure. These results provide mechanistic insight into the factors regulating AT2 cell stemness during health and CS-associated diseases.

We previously reported that long-term intermittent CS exposure enhanced tumorigenesis and emphysema progression when compared to continuous CS exposure [6]. Aberrant proliferation is a major component of cancer development and progression [36]. Cancer develops when a single mutated cell begins to proliferate abnormally. AT2 cells are widely held to be the cell of origin in lung cancer. Using lineage tracing, Desai et al. demonstrated that an activating Kras mutation in AT2 cells initiated a tumor focus [37]. In our previous long-term CS-induced lung tumor model, tumor cells were identified to originate from AT2 cells in the intermittent CS-exposed group based on their uniform Sftpc-positive and club cell 10-kD protein (CC10)-negative state [6]. A notable finding of the current study was that even short-term intermittent CS exposure enhanced the self-proliferation capacity of AT2 cells and blocking FAO reversed this enhancement. More studies on the role of FAO in lung cancer initiation and its relationship to the pattern of CS exposure are warranted.

## Conclusion

In conclusion, alveolar stem cell function was more strongly activated by intermittent CS exposure than by continuous CS exposure. We successfully demonstrated that intermittent CS exposure enhanced the stemness of AT2 cells via FAO activation. These results provide mechanistic insight into the factors regulating AT2 cell stemness during health and CS-associated diseases.

## Supplementary Information

**Additional file 1.** Supplemental Figure S1.

**Additional file 2.** Supplemental Figure S2.

**Additional file 3.** Supplemental Figure S3.

**Additional file 4.** Supplemental Figure S4.

**Additional file 5.** Supplemental Table S1.

## Data Availability

The data that support the findings of this study are available from the corresponding author upon reasonable request.

## Abbreviations

ACK: Ammonium-chloride-potassium
Aqp5: Aquaporin-5
AT1 cell: Alveolar type 1 cell
AT2 cell: Alveolar type 2 cell
BADJ: Bronchioalveolar duct junction
BAL: Bronchoalveolar lavage
BALF: Bronchoalveolar lavage fluid
BMP4: Bone marrow protein 4
CC10: Club cell 10-kD protein
CFE: Colony forming efficiency
COPD: Chronic obstructive pulmonary disease
Cpt1a: Carnitine palmitoyltransferase 1a
CS: Cigarette smoke
EpCAM: Epithelial cell adhesion molecule
FACS: Fluorescence-activated cell sorting
FAO: Fatty acid oxidation
FBS: Fetal bovine serum
GFP: Green fluorescent protein
GSH: Glutathione
GSSG: Glutathione disulfide
H&E: Hematoxylin and eosin
SD: Standard deviation
Sftpc: Surfactant protein C
WLC: Whole lung cell
3D: Three-dimensional
8-OHdG: 8-Hydroxy-2′-deoxyguanosine
