# Supplementary figures and images for: Short-term intermittent cigarette smoke exposure enhances alveolar type 2 cell stemness via fatty acid oxidation
Source: Respir Res. 2022 Mar 2;23:41. doi: 10.1186/s12931-022-01948-4 (PMC8889685; doi:10.1186/s12931-022-01948-4)

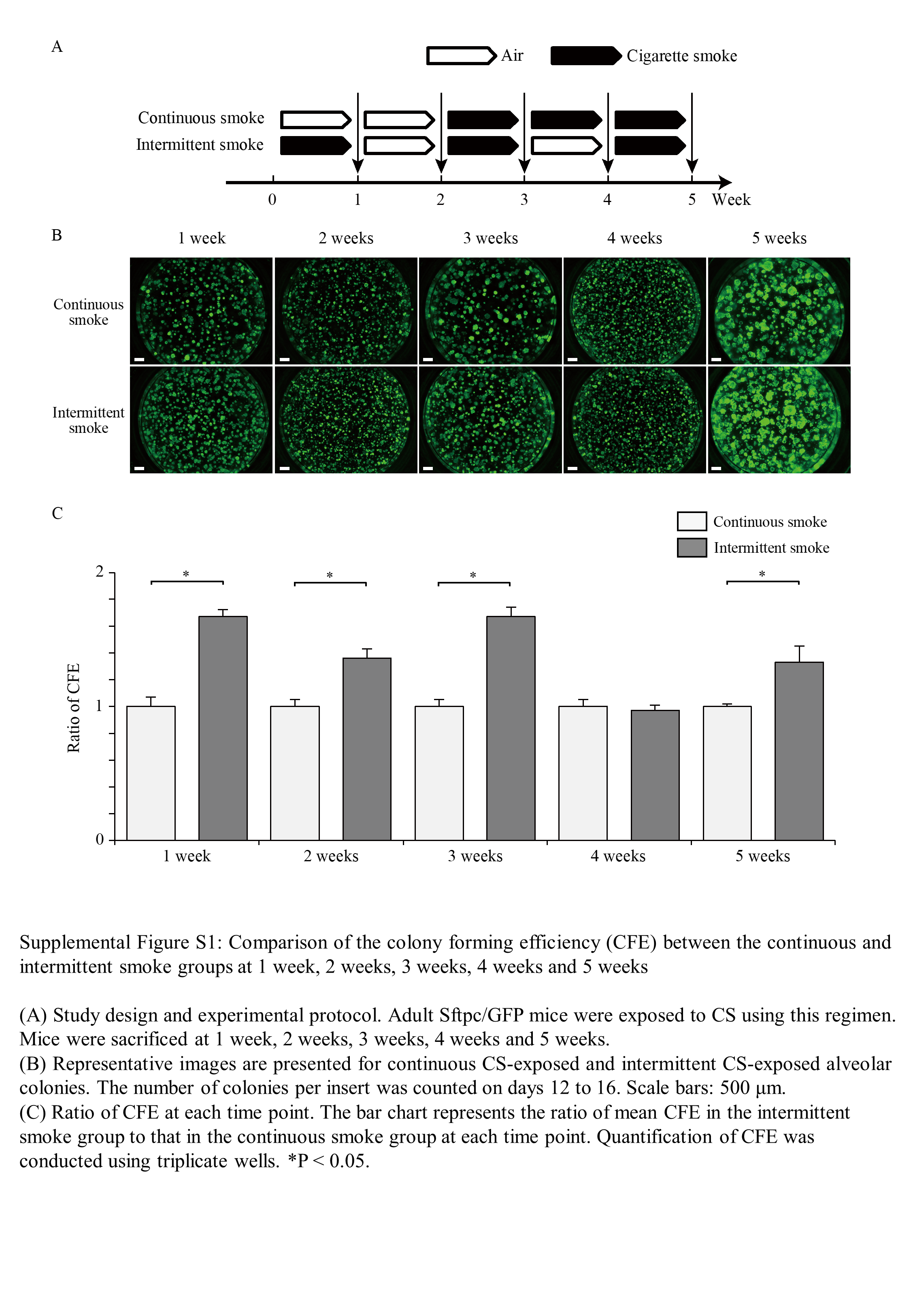

Supplement: Supplementary file 1 — Additional file 1. Supplemental Figure S1. [file 12931_2022_1948_MOESM1_ESM.tif]

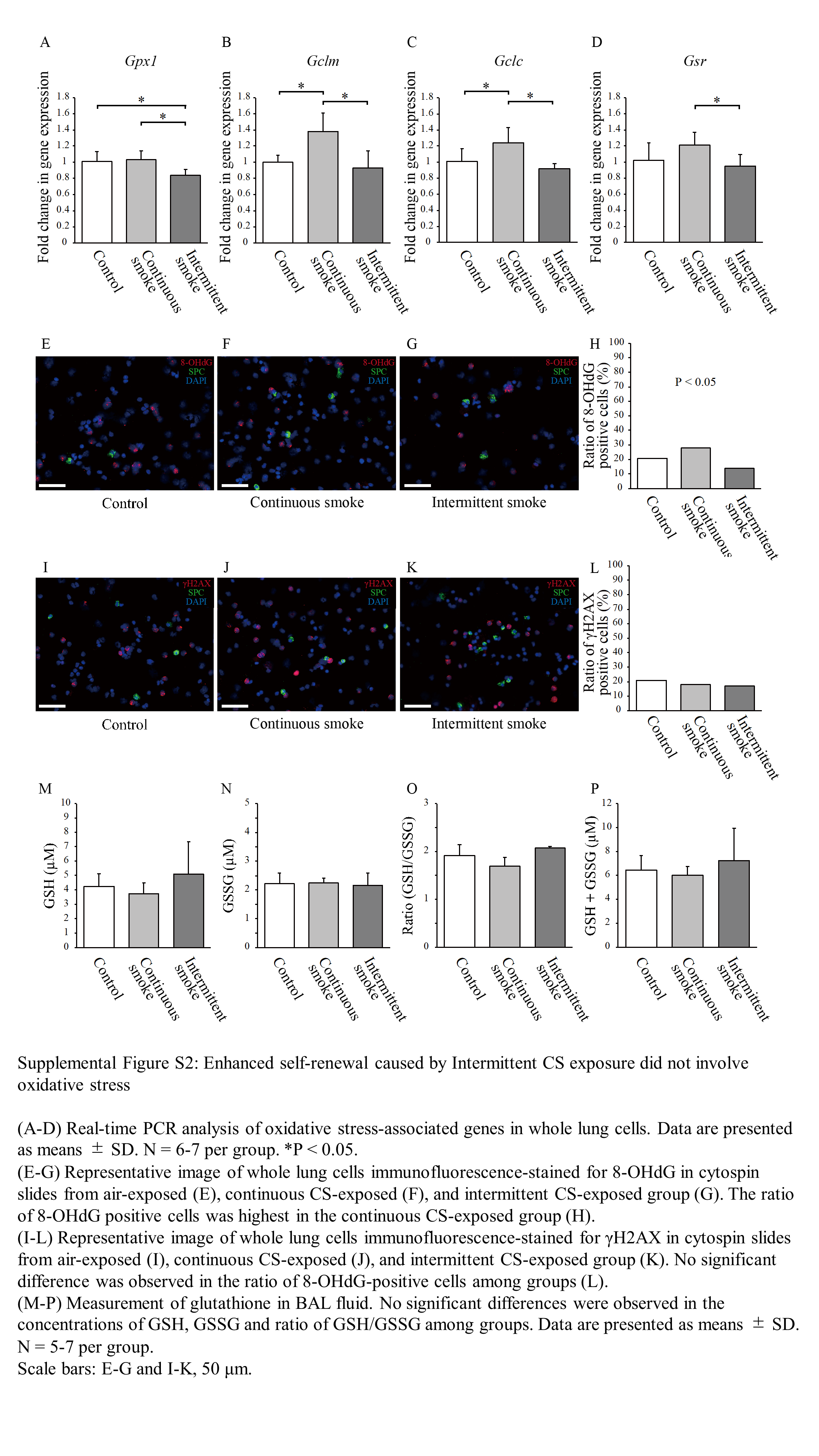

Supplement: Supplementary file 2 — Additional file 2. Supplemental Figure S2. [file 12931_2022_1948_MOESM2_ESM.tif]

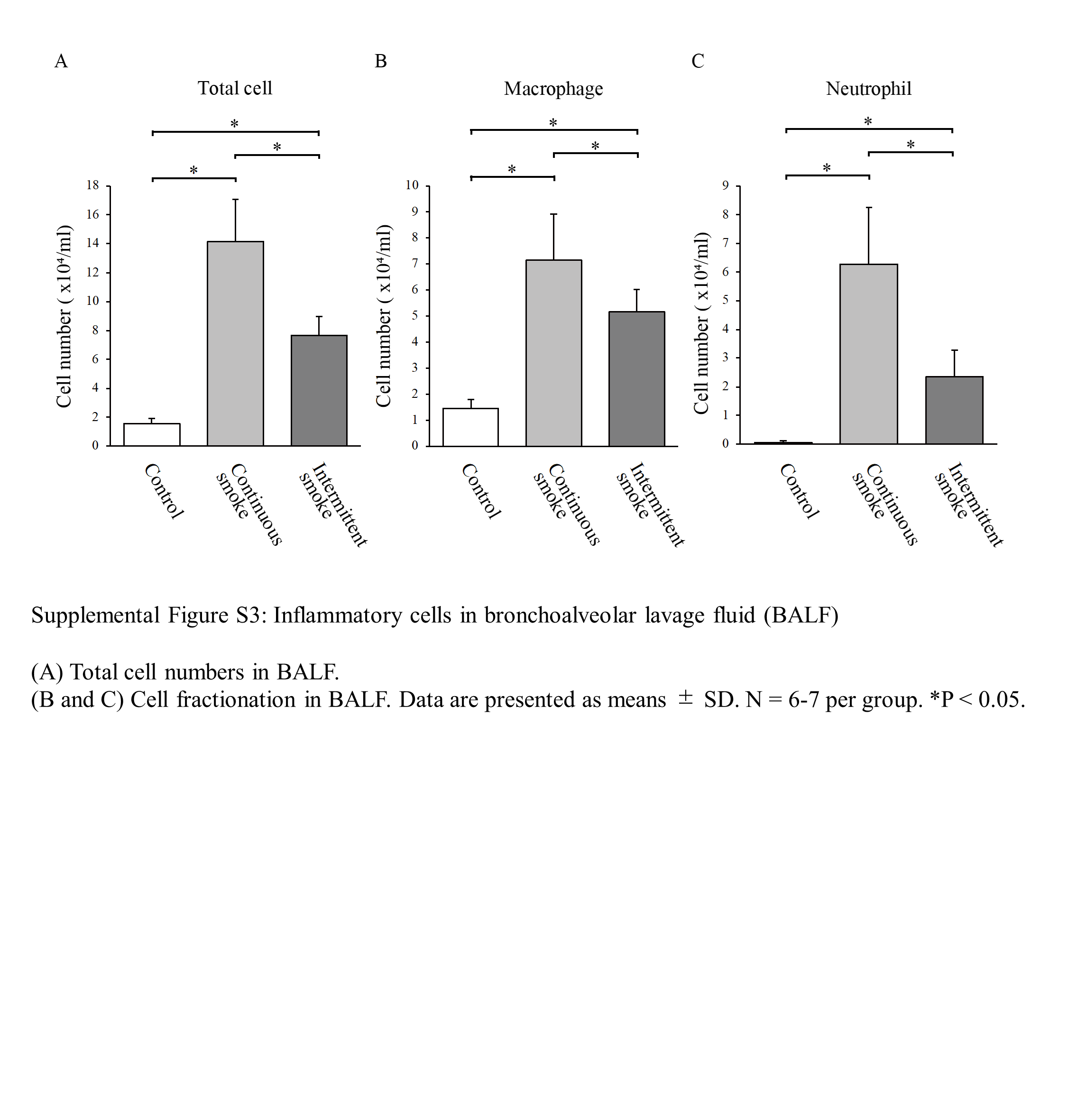

Supplement: Supplementary file 3 — Additional file 3. Supplemental Figure S3. [file 12931_2022_1948_MOESM3_ESM.tif]

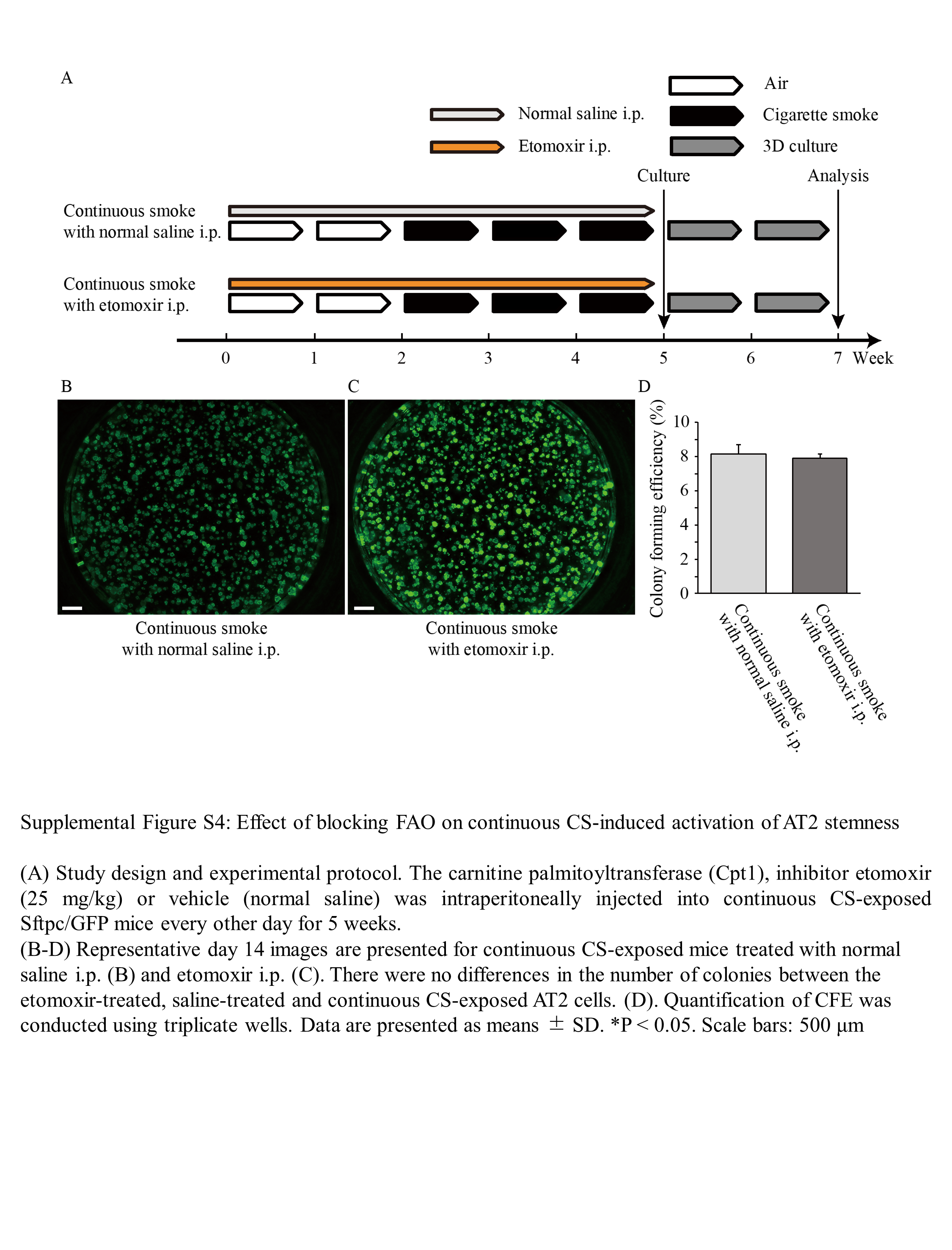

Supplement: Supplementary file 4 — Additional file 4. Supplemental Figure S4. [file 12931_2022_1948_MOESM4_ESM.tif]

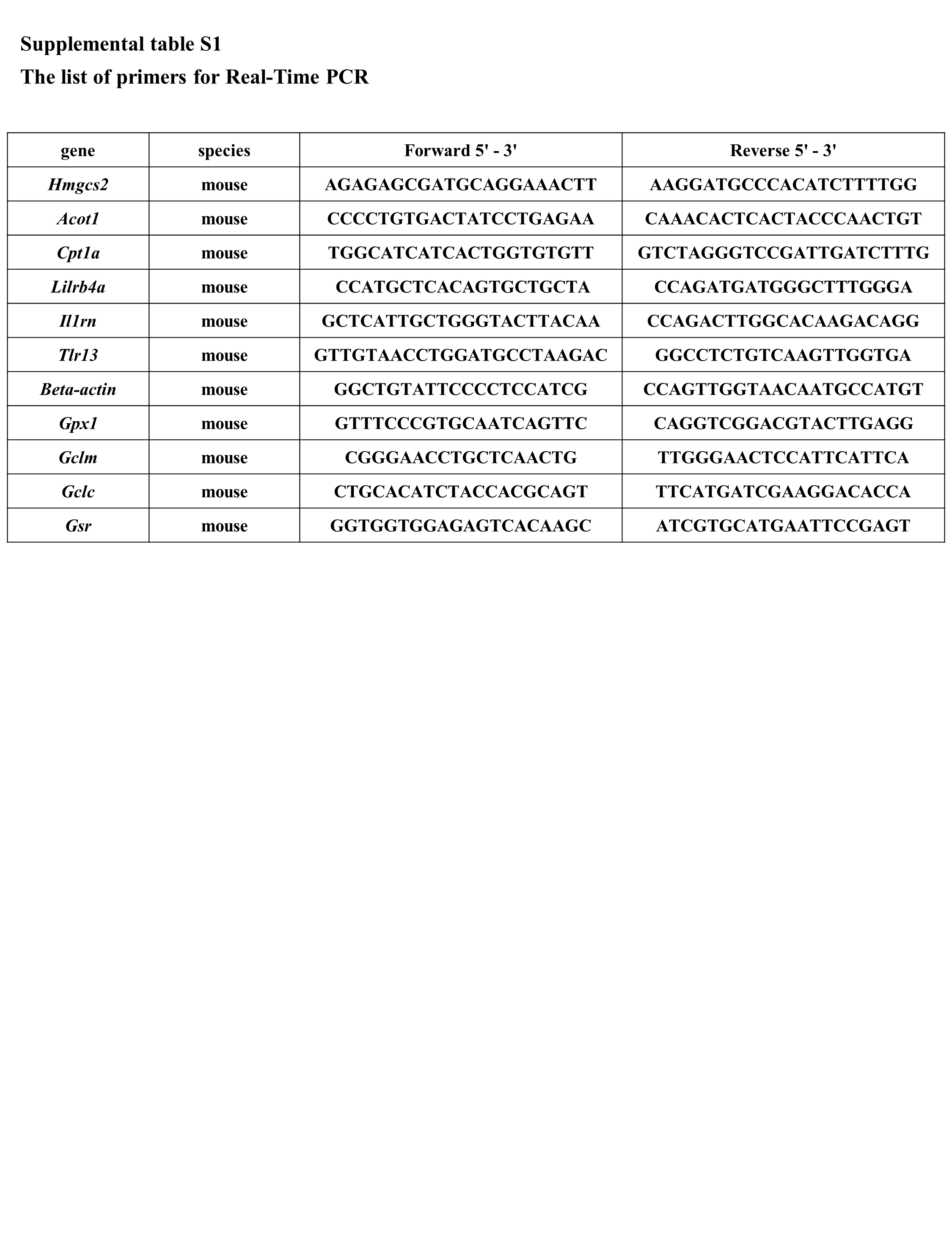

Supplement: Supplementary file 5 — Additional file 5. Supplemental Table S1. [file 12931_2022_1948_MOESM5_ESM.tif]
